# Butyric Acid Ameliorates Myocardial Fibrosis by Regulating M1/M2 Polarization of Macrophages and Promoting Recovery of Mitochondrial Function

**DOI:** 10.3389/fnut.2022.875473

**Published:** 2022-05-18

**Authors:** Xiaogang Li, Ruixuan Li, Nana You, Xiexiong Zhao, Jiaying Li, Weihong Jiang

**Affiliations:** Department of Cardiology, The 3rd Xiangya Hospital of Central South University, Changsha, China

**Keywords:** butyric acid, myocardial fibrosis, macrophage polarization, mitochondria, gut microbiota

## Abstract

**Background:**

We aimed to investigate the effect and mechanism of butyric acid on rat myocardial fibrosis (MF).

**Methods:**

16S rRNA sequencing was used to analyze the gut microbiota characteristics of the Sham group and MF group. HPLC was applied to measure butyric acid in the feces and serum. *In vitro*, rat macrophages RMa-bm were stimulated with LPS and IL-4, respectively, and then butyrate was added to study the influences of butyrate on M1/M2 polarization and mitochondrial function of rat macrophages. The rat macrophages and rat myocardial fibroblasts were co-cultured to explore the effect of butyrate on rat myocardial fibroblasts. In addition, MF rats were fed with butyric acid diet.

**Results:**

Compared with the Sham group, collagen deposition in the MF group was increased, and fibrosis was serious. The abundance of *Desulfovibrionaceae* and *Helicobacteraceae* in the MF group was increased compared with the Sham group. Gut epithelial cells were destroyed in the MF group compared with the Sham group. Compared with the Sham group, LPS content in the MF group was increased and butyric acid was decreased. Butyrate inhibited M1 and promoted M2. Furthermore, butyrate may promote mitochondrial function recovery by regulating M1/M2 polarization of macrophages. After adding butyrate, cell proliferation ability was decreased, and aging and apoptosis were increased, which indicated that butyrate inhibited rat myocardial fibroblasts activity. Moreover, butyric acid could protect mitochondria and improve the symptoms of rats with MF.

**Conclusions:**

Butyric acid ameliorated MF by regulating M1/M2 polarization of macrophages and promoting recovery of mitochondrial function.

## Introduction

Myocardial fibrosis (MF) refers to various quantitative and qualitative changes in the myocardial interstitial collagen network because of cardiac ischemia, global diseases, drugs or any other harmful stimulation that affects the circulatory system or the heart itself (1). It is mainly manifested by the proliferation of myocardial fibroblasts, which secretes extracellular matrix proteins to replace damaged tissues (2). It is a common pathological manifestation in the end stage of many cardiovascular diseases and is the result of imbalance of collagen synthesis and metabolism (3, 4). MF might reflect the activation of repair or maladaptive processes (5). When MF occurs, it will damage the myocardial structure and promote arrhythmia and ischemia, thus affecting the evolution and outcome of heart disease (6). The treatments to alleviate MF have yet to be developed to effectively protect the heart.

Recent studies have shown that gut microbiota has a variety of effects on the host. Indeed, the functions of gut microbiota like an endocrine organ, producing bioactive metabolites that affect the physiological function of the host (7). There is growing awareness of the importance of the gut in many cardiovascular health and diseases, of which the role of the “gut-heart” axis is particularly important. Decreased cardiac function could reduce intestinal perfusion and lead to morphological changes, which may lead to changes in the composition of gut microbiota in patients with heart failure (8). Butyric acid is a short-chain fatty acid (SCFA) from the microbial community that involves in a series of cellular processes in a concentration-dependent manner (9). It is a multifunctional molecule produced by the fermentation of dietary fiber in the intestinal tract of mammals and is rich in butter and other dairy products (10). Butyrate reduces lipopolysaccharide (LPS) damage to intestinal barrier integrity and tight junction permeability in a dose-dependent manner, while LPS leakage intensifies oxidative stress and inflammatory activities related to increased intestinal permeability (11, 12). Obviously, this is important for the “gut-heart” axis function. Butyrate could also pass through intestinal epithelial cells and enter systemic circulation, thereby weakening the immune response and better optimizing mitochondrial function (13). However, the exact mechanism of the mitochondrial protective function provided by butyric acid remains unknown.

Macrophages are key cells in the immune inflammatory response. Activated macrophages are generally differentiated into M1 and M2 phenotypes (14). In addition to playing a role in host defense, macrophages also ensure tissue homeostasis and inhibit inflammatory responses. In order to perform these seemingly opposite functions, macrophages show high plasticity and adopt a spectrum of polarized states, where M1 macrophages and M2 macrophages are extreme (15, 16). Both M1 macrophages and M2 macrophages are closely related to the inflammatory response, among which M1 macrophages are mainly involved in the pro-inflammatory response, and M2 macrophages are mainly involved in the anti-inflammatory response (17). Functionally, M2 macrophages inhibit M1-driven inflammation and promote tissue repair. Ameliorating the inflammatory environment by modulating the activation state of macrophages is an effective approach for the treatment of disease. The repolarization of macrophages is a novel therapeutic approach. For example, the treatment of chronic inflammatory diseases such as atherosclerosis and rheumatoid arthritis will benefit from the repolarization of inflammatory M1 macrophages into anti-inflammatory M2 macrophages (18–20). It has been reported that the mitochondrial metabolism of primary microglia and mouse BV-2 microglia changes under LPS (M1) and IL-4/IL-13 (M2) polarization (21). However, the specific mechanism leading to this effect remains unresolved in MF.

Based on the above background, we hypothesized that M1-related mitochondrial oxidative phosphorylation inhibition is the factor that prevents M1 from repolarization to M2. Increasing the plasma concentration of butyric acid helps to protect the mitochondria, thereby repolarizing M2 from M1 to inhibit the progression of MF. To this end, we constructed a rat model of MF and fed rats with butyric acid. 16S rRNA sequencing was used to analyze the gut microbiota characteristics of the Sham group and MF group. In addition, influences of butyrate on M1/M2 polarization of rat macrophages, mitochondrial function and rat myocardial fibroblasts were investigated *in vitro* experiments. Our study provides an important clue for further understanding the mechanism of the occurrence and development of MF, and also provides a reference and basis for the clinical treatment of MF.

## Materials and Methods

### Animal

According to previous reports, transverse aortic constriction (TAC) surgery was performed to induce cardiac pressure overload (22). A total of 24 male SD rats with similar body weight were randomly divided into three groups: Sham group, MF group, and Butyrate group, with 8 rats in each group. Sham group was used as the control group. Briefly, the MF group was treated with lateral thoracotomy in the second intercostal space under inhalation anesthesia (1.2% vol isoflurane, 70% N_2_O/30% O_2_) without entering the thoracic cavity. Polypropylene suture 6.0 was used to narrow the brachiocephalic artery. The brachiocephalic artery and the left common carotid artery were narrowed by using polypropylene 6.0 suture at 27 g needle interval. For the Sham group, we followed the same protocol, except that the aorta was not ligated. Intercostal space and skin were sutured with polypropylene 6.0. All further experiments were performed 14 days after TAC surgery (13). The echocardiography, ejection fractions (EF), left ventricular fractional shortening (FS), left ventricular posterior wall diameter in diastole (LVPWd), and left ventricular posterior wall diameter in systole (LVPWs) were examined. The Butyrate group was treated as follows: the rats were fed with 0.63 g/kg butyric acid (99% purity) for 8 days, and the indexes were determined after 8 days (23). Mice were sacrificed by intraperitoneal injection of sodium pentobarbital (150 mg/kg). All animal experiments were approved by the IRB of Third Xiangya Hospital, Central South University (2018-S259). All methods were carried out in accordance with the relevant guidelines and regulations.

### Masson Staining

The rat heart tissues were taken to make paraffin sections. The slices were roasted at 60°C for more than 3 h, and dewaxed to water. The appropriate amount of nuclear dye was added to cover the whole tissues, and stained for 3–5 min. We rinsed the staining solution completely with tap water, soaked the slices with distilled water, and then soaked the slices with weak alkaline solvent such as PBS or ammonia for 5–10 min to make the nuclei blue again. We removed the water from the slices, dropped the dye to cover the whole tissues, stained them for 6–8 min, and rinsed the dye with rinse solution. The samples were sealed with neutral gum. The results were observed under a microscope (BA210T, Motic, China).

### 16S rRNA Sequencing

Fecal samples from the Sham group and MF group were collected to detect microbial diversity. The V3-V4 region of 16S rRNA gene was sequenced using the following 16S rRNA gene V3-V4 region-specific primers: 341F, CTACGGGNGGCWGCAG, and 805R, GACTACHVGGGTATCTAATCC. Illumina NovaSeq PE250 was used for 16S amplicon sequencing and the raw data were obtained. Qiime2 (QIIME2-2020.2) and R software (4.0.2) were used for sequence data analysis. After obtaining the original data for quality control, on the one hand, species composition in samples was analyzed by comparing species database; On the other hand, assembly, gene prediction, and functional annotation were performed.

### HE Staining

The gut and heart tissues of rats were taken, fixed with formalin, and made into paraffin sections. The slices were baked in a 60°C microwave oven for 2 h. Then the slices were placed in xylene for 15 min, three times. Gradient alcohol was then added and placed for 5 min each. Then they were soaked in distilled water for 5 min. After dyeing with hematoxylin for 3 min, the slices were rinsed with distilled water and returned to blue with PBS. After staining with eosin for 5 s, the slices were rinsed with distilled water and dehydrated with graded alcohol (95-100%) for 5 min per grade. After taking them out, they were placed in xylene for 10 min, sealed with neutral gum and observed under a microscope (BA210T, Motic, China).

### Enzyme Linked Immunosorbent Assay (ELISA)

The levels of LPS, IL-6, TNF-α, IL-12, 8-Oxo-dG, ATP, and Citrate synthase (CS) were measured in the plasma and myocardial tissues of rats. LPS (CSB-E14247R, CUSABIO), IL-6 (CSB-E04640R, CUSABIO), TNF-α (CSB-E11987R, CUSABIO), IL-12 (CSB-E07362R, CUSABIO), 8-oxo-dG (ML059056, MLBIO), ATP (A095-1-1, Nanjing Jiancheng Bioengineering Institute), and CS (A108, Nanjing Jiancheng Bioengineering Institute) assay kit were applied to detect LPS, IL-6, TNF-α, IL-12, 8-oxo-dG, ATP, and CS levels according to the introductions. The concentration of LPS, IL-6, TNF-α, IL-12, 8-oxo-dG, ATP, and CS was calculated by using the standard curve provided by DYNATECHMR7000 micrometer.

### High-Performance Liquid Chromatography-Mass Spectrometry (HPLC-MS)

Determination of r-aminobutyric acid in the plasma and feces were detected by HPLC-MS. 1 mL r-aminobutyric acid were weighed, then 9 mL acetonitrile was added, vortexed for 1 min, and centrifuged at 10,000 r/min for 10 min. The supernatant solution was applied for HPLC analysis (LC-30, Shimadzu, Tokyo, Japan). The mother liquor of standard substance was taken, and water was added to prepare standard substance solutions with the concentration of 10, 20, 50, 100, and 200 ng/mL, respectively. The solution was filtered through 0.22 μm membrane and analyzed by HPLC-MS. The liquid chromatography was performed on Waters ACQUITY UPLC column with BEH amide (100 × 2.1 mm, 1.7 μm). Mobile phase was: A: 0.2% formic acid aqueous solution, B: Acetonitrile. Column temperature was 35°C. Flow rate was 0.2 mL/min, and injection volume was 1 μL. Electrospray ionization (ESI) positive ion mode was used for detection. Scanning mode was multiple reaction monitoring (MRM). Dry gas was 10.0 L/min nitrogen. Heating gas was 10.0 L/min air. Collision gas was 270 kPa argon. Atomization gas was 3.0 L/min. Interface temperature was 200°C. DL tube temperature was 280°C. Dwell time was 150 ms. Heating module temperature was 300°C. Delay time was 3 ms, and interface voltage was 1.0 kV.

### Cell Culture and Treatment

The rat macrophage line RMa-bm was purchased from American type culture collection (ATCC, USA) and cultured in the DMEM medium containing 10% fetal bovine serum and 1% penicillin/streptomycin. Rat myocardial fibroblasts were purchased from BeNa Culture Collection (#BNCC341875, China) and cultured in DMEM medium with high glucose and 10% fetal bovine serum. Culture environment was at 37°C and 5% CO_2_. RMa-bm was inoculated and cultured at 2 × 10^6^/mL during the experiment. In order to further explore the influence of butyrate on M1 polarization, RMa-bm were stimulated with LPS for 24 h, and then butyrate was added. Groups were as follows: Control group (RMa-bm), LPS group (100 ng/mL LPS stimulated RMa-bm for 24 h), LPS+butyrate group (RMa-bm were stimulated by 100 ng/mL LPS and then 1 mM butyrate was added). In order to study whether butyrate involved in M2 polarization, RMa-bm were treated with IL-4 for 24 h, and then butyrate was added. They were grouped into the Control group (RMa-bm), IL-4 group (20 ng/mL IL-4 stimulated RMa-bm for 24 h), and IL-4+butyrate group (RMa-bm were stimulated by 20 ng/mL IL-4 and then 1 mM butyrate was added) (15). The co-culture method of RMa-bm and myocardial fibroblasts (1:1) was as follows: RMa-bm was inoculated in Transwell subchamber with 2 × 10^6^/mL. The experiment was randomly divided into five groups: Control group (RMa-bm co-cultured with myocardial fibroblasts), LPS group (RMa-bm were stimulated by LPS and co-cultured with myocardial fibroblasts), LPS+butyrate group (RMa-bm were stimulated by LPS and 1 mM butyrate was added and then co-cultured with myocardial fibroblast), IL-4 group (RMa-bm were stimulated by IL-4 and co-cultured with myocardial fibroblasts), IL-4+butyrate group (RMa-bm were stimulated by IL-4 and 1 mM butyrate was added and then co-cultured with myocardial fibroblast).

### Flow Cytometry

The positive cell rates of CD86 (+), CD80 (+), CD64 (+), CD16/32(+), CD206 (+), and CD163 (+) were determined as follows: 1 × 10^6^/100 μL cells were placed into a 1.5 mL EP tube. 1 mL PBS was added to wash the cells, and centrifuged at 350 g for 5 min, and then the supernatant was discarded. Another 1 mL PBS was added for washing once, centrifuged at 350 g for 5 min, and the supernatant was discarded. Antibodies CD86 (11-0862-820, ebioscience), CD80 (12-0800-82, ebioscience), CD64 (orb124535, biorbyt), CD16/32 (12-0161-82, ebioscience), CD206 (12-2061-82, ebioscience), and CD163 (11-1631-82, ebioscience) were added to each tube, and stained for 30 min at room temperature without light. 1 mL PBS was added to wash cells, centrifuged at 350 g for 5 min, and then the supernatant was discarded. 150 μL PBS was resuspended for precipitation, and detection was performed on the flow cytometry (A00-1-1102, Beckman, USA). The positive cell rates of TNF-α (+) and IL-10 (+) were detected as follows: 1 × 10^6^/100 μL cells were placed into a 1.5 mL EP tube, and the volume was increased to 500 μL. 1 μL cell stimulation cocktail (Plus protein transport inhibitors) was added. They were cultured in 37°C constant temperature incubator for 4 h and centrifuged at 350 g. 1 mL PBS was added to wash the cells, centrifuged at 350 g for 5 min, and the supernatant was discarded. Another 1 mL PBS was added for washing once, centrifuged at 350 g for 5 min, and then the supernatant was discarded. Fixation/Permeabilization concentrate was diluted into 1 × working solution by 1:3 in Fixation/perm Diluent. The cell precipitates were suspended in the 500 μL 1 × working solution, fixed and broken at room temperature without light for 30 min. Then they were centrifuged at 350 g for 5 min, and the supernatant was discarded. 10 × Permeabilization Buffer was diluted to 1 × working solution with deionized water at 1:9. 1 mL 1 × working solution was added to the above cell precipitate. The cells were re-suspended, centrifuged at 350 g for 5 min, and the supernatant was discarded. 1 mL 1 × working solution was added for repeated washing, centrifuged at 350 g for 5 min, and the supernatant was discarded. The cell precipitates were suspended with 100 μL PBS, and corresponding antibodies TNF-α (0.25 μg) and IL-10 (0.25 μg) were added, respectively. The cells were mixed and incubated at room temperature without light for 30 min. 1 mL PBS was added to wash the cells once, centrifuged at 350 g for 5 min, and the supernatant was discarded. The cell precipitates were suspended with 150 μL PBS and detected by the flow cytometry (A00-1-1102, Beckman, USA).

### JC-1 Staining

Mitochondrial membrane potential assay kit with JC-1 (C2006, Beyotime, China) was used to observe the entire cell morphology and compare the changes of mitochondrial membrane potential between different groups. 50 μL JC-1 (200 ×) was diluted with 8 mL ultrapure water, and then added 2 mL JC-1 staining buffer (5 ×), the mixture was JC-1 staining working solution. 1 mL JC-1 staining working solution was added to the culture plate and incubated in the cell culture box at 37°C for 20 min. After incubation, they were washed twice with JC-1 dyeing buffer (1 ×) and photographed for observation.

### Cell Counting Kit 8 (CCK-8) Assay

The cells were digested with trypsin digestion solution to prepare cell suspension, which was inoculated into 96-well-plates according to the ratio of 5 × 10^3^. Each group was set with 3 multiple wells for corresponding intervention treatment according to the experimental groups, and cultured for 24 h. After corresponding time of cultivation, the medium was discarded and replaced with 10 μL CCK-8 working solution. The medium was incubated for 4 h in an incubator with 5% CO_2_ at 37°C. The absorbance was measured at 450 nm using Elx800 (BioTek, Winooski, Vermont, USA). The proliferation ability of cells was detected at 24, 48, and 72 h.

### Clone Formation Assay

The exponential growth stage cells were taken and each well was seeded with 200 cells in a 6-well-plate containing 1 mL culture medium restored to the room temperature. The cells were placed in a 37°C, 5% CO_2_ and saturated humidity incubator for 2 to 3 weeks, during which liquid was appropriately changed. The culture medium was discarded, PBS solution was carefully immersed, and 1 mL 4% paraformaldehyde was added to each well to fix the cells for 15 min. Then the fixing solution was removed, 1 mL dye solution was added to the working solution and stained for 30 min at room temperature. After depolarization, the absorbance value at 550 nm was determined by enzyme-plate reader.

### Senescence-Associated Beta-Galactosidase (SA-β-Gal) Staining

The 12-well-plate was taken out, the cell culture medium was sucked out. Then cells were washed with PBS once, and 1 mL β-galactosidase staining fixative was added, fixed at room temperature for 15 min. The cell fixative solution was aspirated and the cells were washed with PBS three times for 3 min each. PBS was drained, 1 mL of staining solution was added to each well-(β-galactosidase staining solution A: 10 μL, β-galactosidase staining solution B: 10 μL, β-galactosidase staining solution C: 930 μL, X-Gal solution 50 μL). Then they were incubated overnight at 37°C. Senescent cells were photographed under an ordinary light microscope.

### Cell Apoptosis

Annexin V-FITC apoptosis detection kit (KGA108, KeyGen, China) was used to detect cell apoptosis. Cells were collected by centrifugation at 1,500 rpm for 5 min and washed by PBS once. 500 μL Binding Buffer was added to suspend cells. 5 μL Annexin V-FITC was added and mixed. Then 5 μL PI was added and mixed. After that, they were reacted at room temperature for 5–15 min. Cell apoptosis was detected by flow cytometry (A00-1-1102, Beckman).

### Quantitative Real-Time PCR (qRT-PCR)

The expression of apoptosis, senescence signaling pathway and inflammatory factors (Bax, Bcl-2, p21, p16, IL-6, IL-12, and TNF-α) were detected by qRT-PCR. Total RNA was extracted by Trizol method and reverse transcribed into cDNAs by cDNA reverse transcription kit (#CW2569, Beijing ComWin Biotech, China). Ultra SYBR Mixture (#CW2601, Beijing ComWin Biotech, China) was utilized to test relative gene expression on ABI 7900 system. With β-actin as the internal genes, the relative levels of were calculated by 2 ^−Δ*ΔCt*^ method. The primer sequences used in this study were shown in Table 1.

**Table 1:** The primers used in this study.

**Name**	**Primers**
Bax-F	TGAAGACAGGGGCCTTTTTG
Bax-R	AATTCGCCGGAGACACTCG
Bcl-2-F	TTGAAAACCGAACCAGGAATTGC
Bcl-2-R	GTCCTGTGCCACTTGCTCT
p21-F	ACTCAACCGTAATATCCCGACT
p21-R	GCAGCAGATCACCAGATTAACCC
p16-F	CAGAGCTAAATCCGGCCTCA
p16-R	CAGTTTCTCATGCCATTCCT
IL-6-F	GACTTCCATCCAGTTGCCTT
IL-6-R	ATGTGTAATTAAGCCTCCGACT
IL-12-F	TGAAGACATCACACGGGACCA
IL-12-R	CAGCTCCCTCTTGTTGTGGAA
TNF-α-F	AGCACAGAAAGCATGATCCG
TNF-α-R	CACCCCGAAGTTCAGTAGACA
IL-10-F	GTTCCCCTACTGTCATCCCC
IL-10-R	AGGCAGACAAACAATACACCA
β-actin-F	ACATCCGTAAAGACCTCTATGCC
β-actin-R	TACTCCTGCTTGCTGATCCAC

### Statistical Analysis

Graphpad Prism8.0 statistical software was used for statistical analysis. Measurement data were expressed as mean ± standard deviation (SD) and repeated at least three times. Student's *t*-test was used between the two groups, and one-way analysis of variance (ANOVA) was used for comparison between multiple groups. Pearson correlation coefficient was used to analyze the correlation between butyric acid and *Helicobacteraceae* and *Desulfovibrionaceae*. *P* < 0.05 indicated that the difference was statistically significant.

## Results

### The Rat MF Model Was Established Successfully

**Figure 1:**
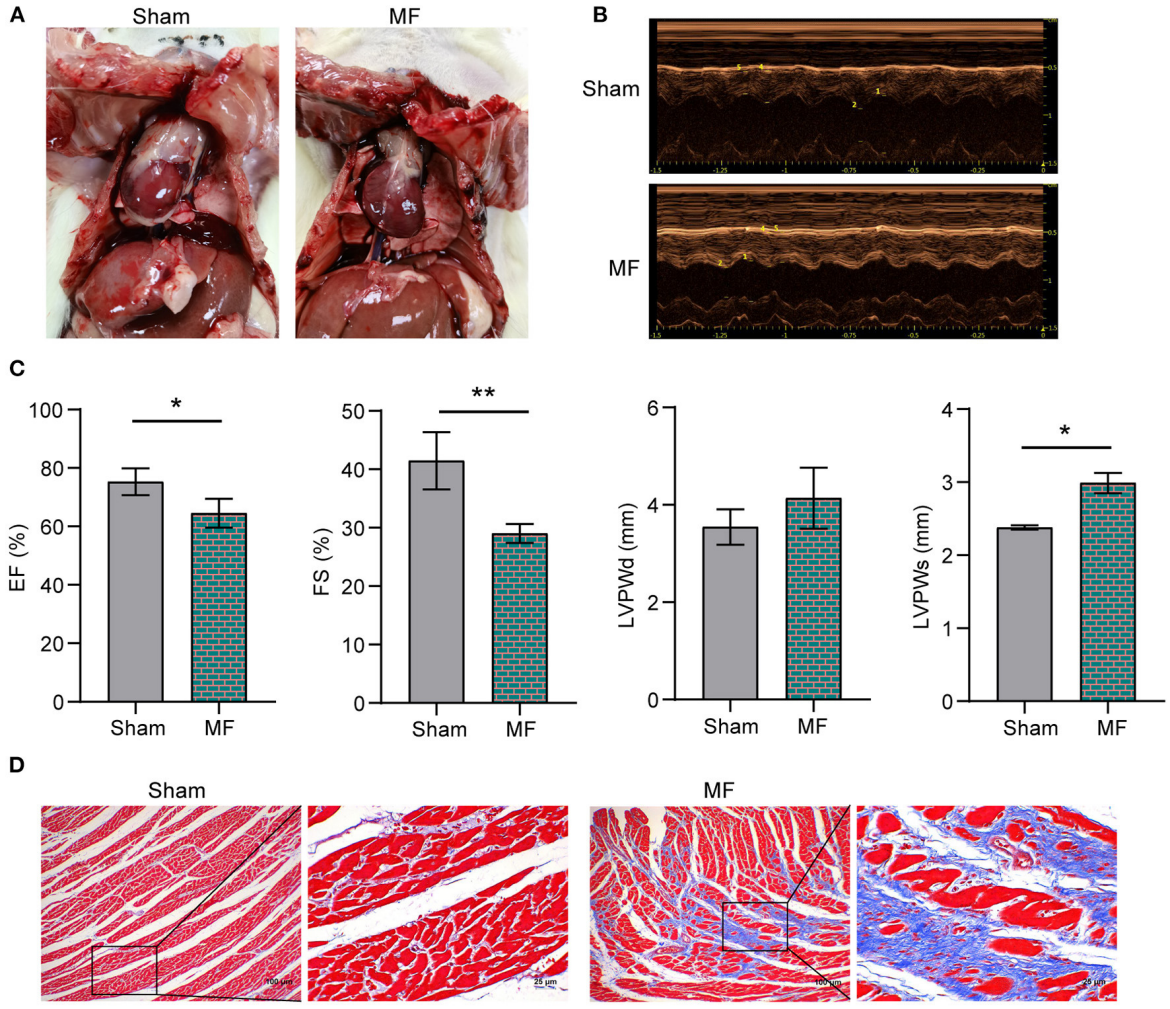
The rat MF model was established successfully. **(A)** Morphology of the rat heart. **(B)** Rat echocardiography. **(C)** EF, FS, LVPWd and LVPWs detection. **(D)** Masson staining was performed to stain the rat heart tissues (×100, 100 μm; × 400, 25 μm). ^*^ *P* < 0.05, **P < 0.01.

Figure 1A showed the morphology of rat heart after modeling. In order to test whether the rat MF model was successfully constructed, echocardiography was performed on the rats first (Figure 1B). Compared with the Sham group, EF, and FS in MF group were decreased. LVPWd and LVPWs were increased (Figure 1C). Masson staining (Figure 1D) showed that compared with the Sham group, collagen deposition content in the MF group was increased and fibrosis was serious. These results indicated that the rat MF model has been established successfully.

### The Content of Butyric Acid Decreased in MF Group

**Figure 2:**
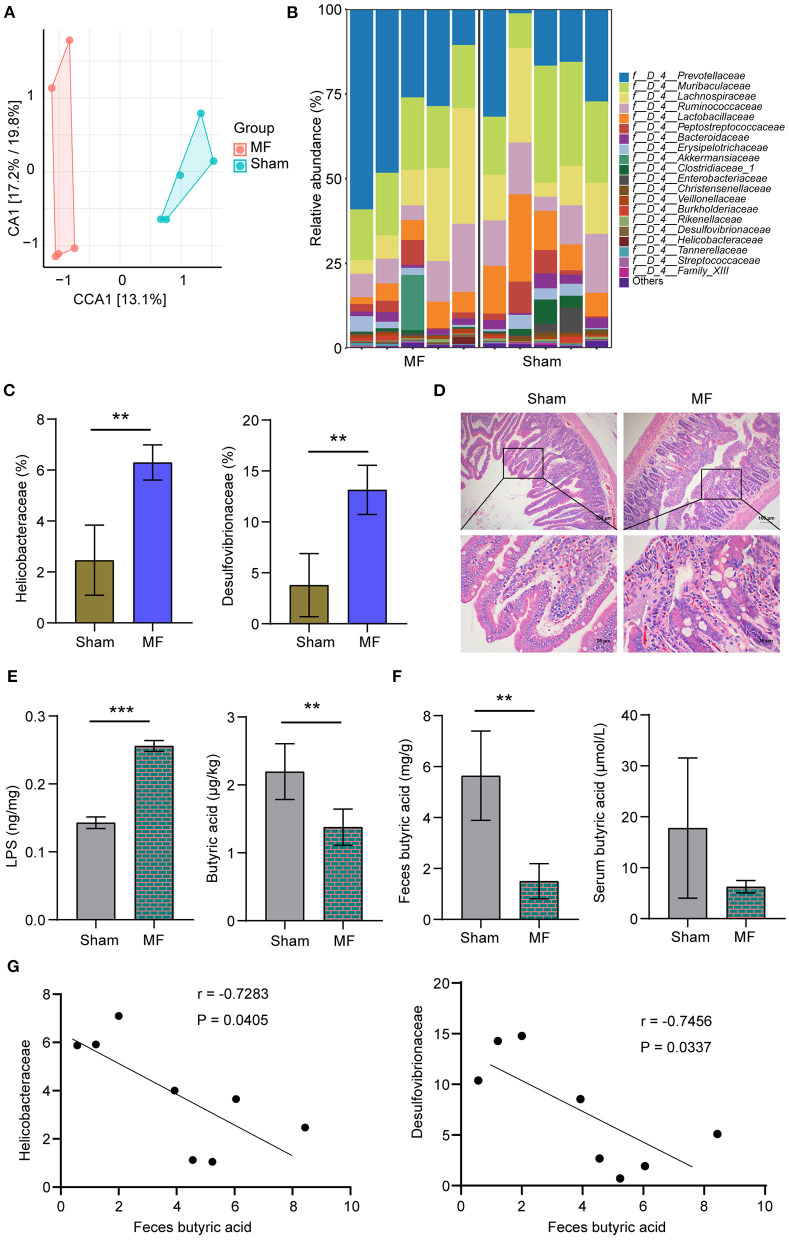
The content of butyric acid was decreased in the MF group. **(A)** PCA of bacterial community distribution. **(B)** Histogram of the relative abundance of top 20 ASVs at the Family level. **(C)** The abundance of *Desulfovibrionaceae* and *Helicobacteraceae*. **(D)** HE staining of the gut (×100, 100 μm; ×400, 25 μm). **(E)** LPS and butyric acid levels in the plasma. **(F)** HPLC detection of butyric acid content in the feces and serum. **(G)** Pearson correlation coefficient analysis between feces butyric acid and *Helicobacteraceae* or *Desulfovibrionaceae*. **P < 0.01, ***P < 0.001.

Next, 16S rRNA sequencing was used to analyze the characteristics of gut microbiota in the Sham group and MF group. Principal component analysis (PCA) of bacterial community distribution in different samples showed significant differences between the two groups (Figure 2A). The histogram of relative abundance of top20 ASVs at the level of Family further showed that the abundance and composition of microbial communities in each group were different (Figure 2B). According to the further analysis, compared with the Sham group, the abundance of *Desulfovibrionaceae* and *Helicobacteraceae* in the MF group were increased (Figure 2C). HE staining showed that compared with the Sham group, gut epithelial cells were destroyed in the MF group (Figure 2D). We also detected the content of butyric acid and LPS in the plasma, and found that compared with the Sham group, LPS content in the MF group increased and butyric acid content decreased (Figure 2E). HPLC results showed that the content of butyric acid in feces and serum of the MF group decreased (Figure 2F). In addition, we performed Pearson correlation coefficient analysis, and the results showed that butyric acid was significantly negatively correlated with *Helicobacteraceae* and *Desulfovibrionaceae* (*P* < 0.05, Figure 2G). Therefore, we were interested in butyric acid changes.

### Influences of Butyrate on M1/M2 Polarization of Rat Macrophages RMa-bm

**Figure 3:**
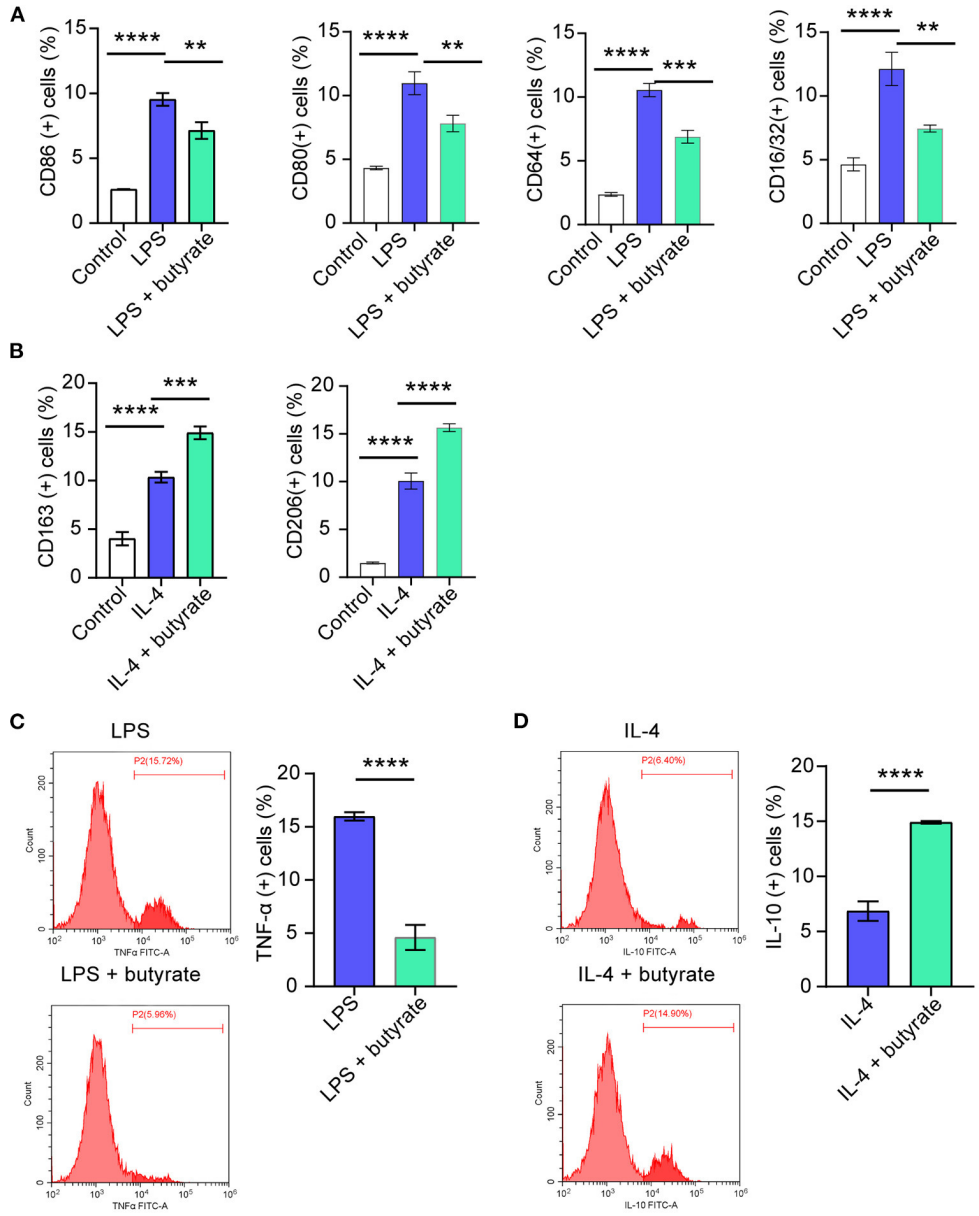
Influences of butyrate on M1/M2 polarization of rat macrophages RMa-bm. **(A–D)**. Flow cytometry was performed to detect CD86 (+), CD80 (+), CD64 (+), CD16/32 (+), CD163 (+), CD206 (+), TNF-α (+), and IL-10 (+) positive cell rates in different treatment groups, respectively. **P < 0.01, ***P < 0.001, ****P < 0.0001.

In order to further explore the influences of butyrate on M1 polarization, rat macrophages RMa-bm were stimulated with LPS for 24 h, and then butyrate was added. Flow cytometry results showed that compared with the Control group, CD86 (+), CD80 (+), CD64 (+), and CD16/32(+) positive cell rates increased in the LPS group, while CD86 (+), CD80 (+), CD64 (+), and CD16/32(+) positive cell rates decreased after butyrate was added (Figure 3A and Supplementary Figure 1A). In addition, after butyrate was added, the positive cell rate of TNF-α (+) was reduced (Figure 3C). To study whether butyrate was involved in M2 polarization, RMa-bm were treated with IL-4 for 24 h, and then butyrate was added. As shown in Figure 3B and Supplementary Figure 1B, compared with the Control group, positive cell rates of CD163 (+) and CD206 (+) in the IL-4 group increased, but after adding butyrate, positive cell rates of CD163 (+) and CD206 (+) increased more obviously. In addition, positive cell rate of IL-10 (+) also increased after butyrate was added (Figure 3D). These data indicated that butyrate suppressed M1 and promoted M2 polarization.

### Butyrate Promoted Mitochondrial Function Recovery by Regulating M1/M2 Polarization of Macrophages

**Figure 4:**
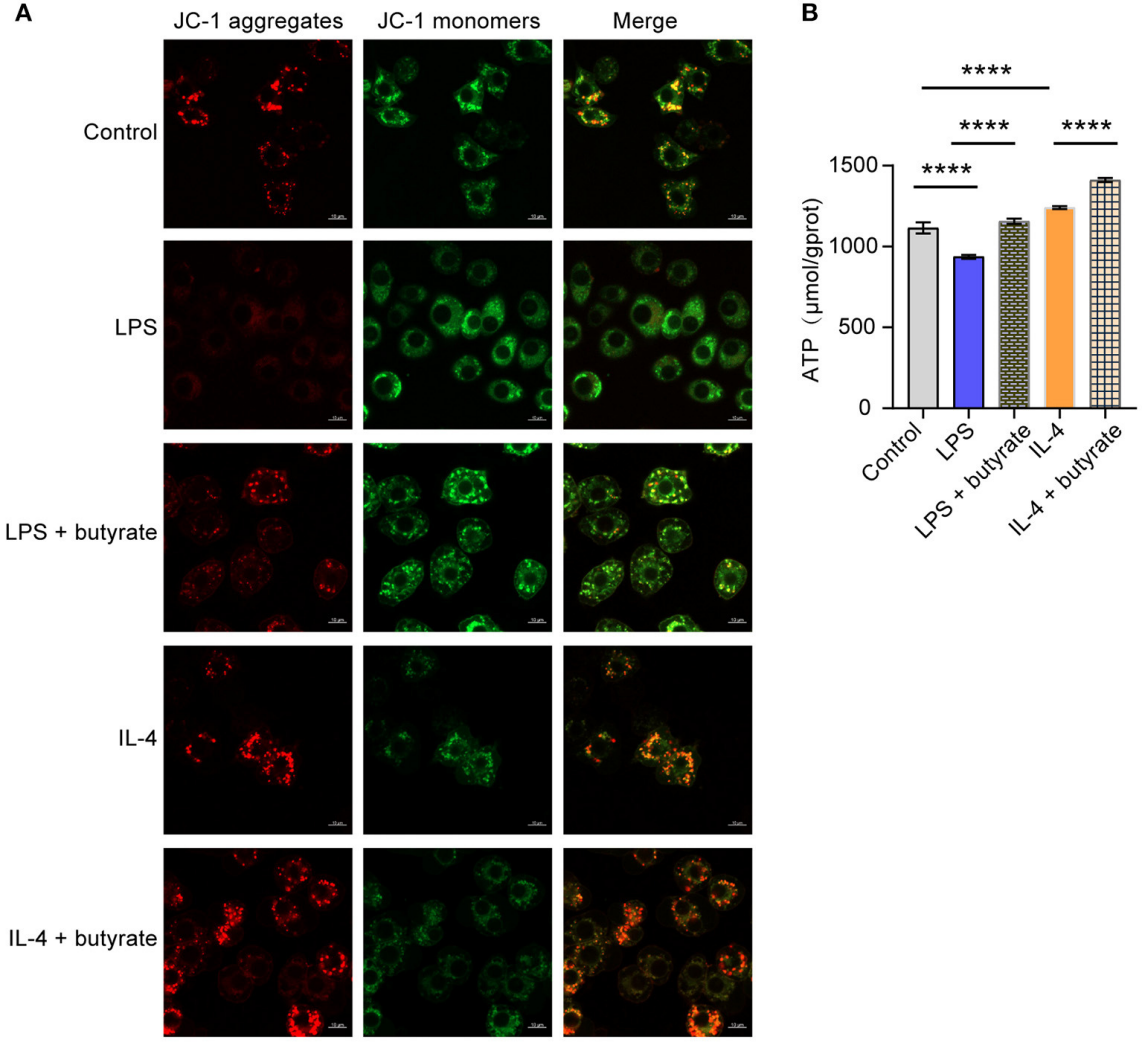
Butyrate regulated M1/M2 polarization of macrophages, thus affecting mitochondrial function. **(A)** Mitochondrial membrane potential detection. **(B)** ATP content. ****P < 0.0001.

Next, we want to explore influences of butyrate on mitochondrial function. We first performed mitochondrial membrane potential detection. Compared with the Control group, mitochondrial membrane integrity was destroyed and membrane potential was lower in the LPS group. The mitochondrial membrane potential of the IL-4 group was high and the membrane was intact. However, after butyrate was added, mitochondrial membrane potential of the LPS+butyrate group and the IL-4+butyrate group was higher and the membrane was intact (Figure 4A). Compared with the Control group, mitochondrial ATP productivity was decreased in the LPS group, but increased in the IL-4 group. However, mitochondrial ATP productivity increased in the LPS+butyrate group and the IL-4+butyrate group (Figure 4B). These results showed that butyrate may promote mitochondrial function recovery by regulating M1/M2 polarization of macrophages.

### Butyrate Inhibited Rat Myocardial Fibroblasts Activity

**Figure 5:**
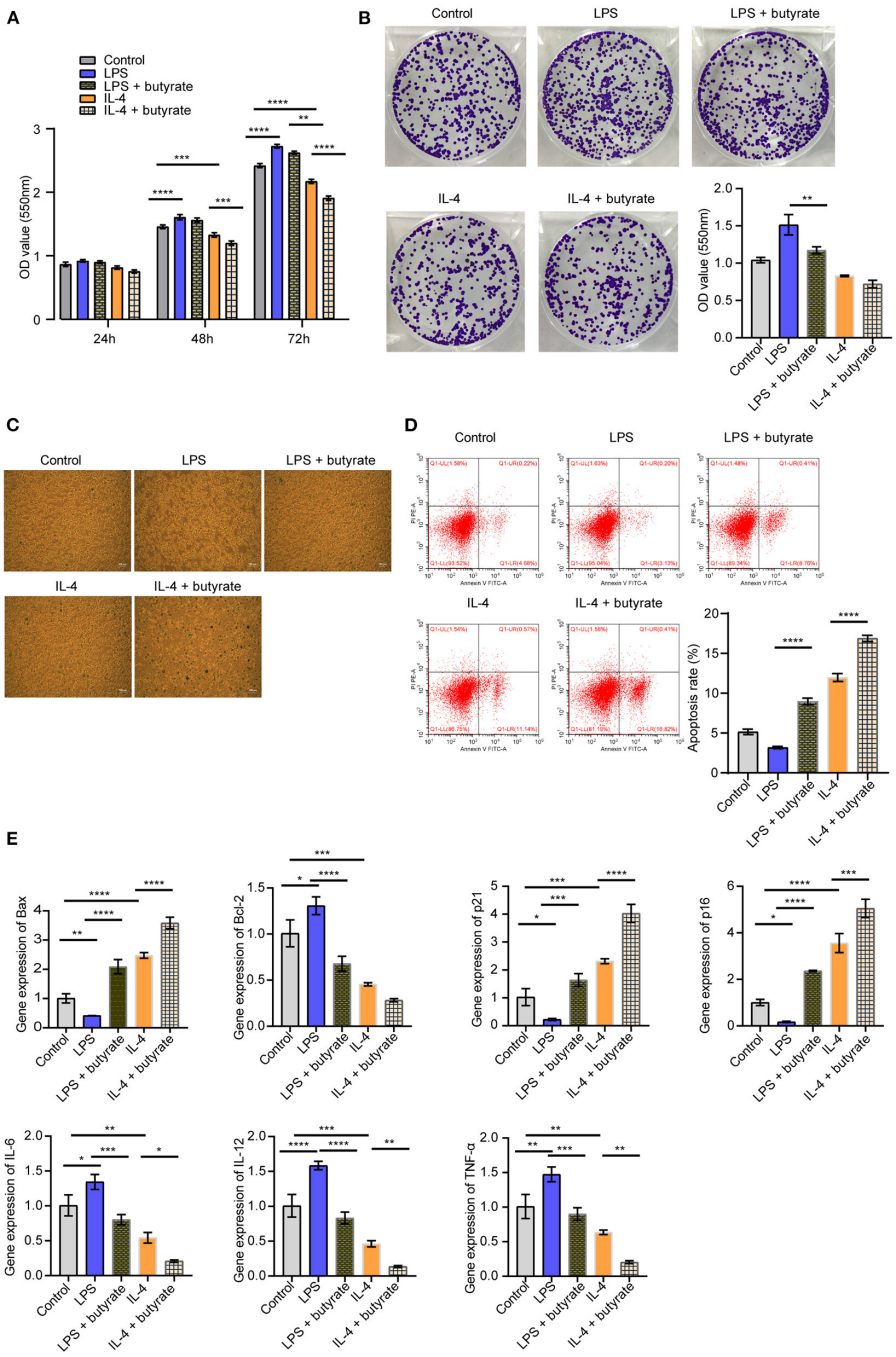
Butyrate inhibited rat myocardial fibroblasts activity. **(A)** CCK-8 was applied to detect cell proliferation. **(B)** Cell proliferation was detected by clone formation assay. **(C)** SA-β-Gal staining was used to detect cell senescence. **(D)** Flow cytometry was used to detect apoptosis. **(E)** The expression of apoptosis, senescence signaling pathway and inflammatory factors (Bax, Bcl-2, p21, p16, IL-6, IL-12, and TNF-α) was measured by qRT-PCR. ^*^P < 0.05, ^**^ *P* < 0.01, ***P < 0.001, ****P < 0.0001.

In order to explore the effects of butyrate on rat myocardial fibroblasts, we conducted co-culture of rat macrophages and rat myocardial fibroblasts. Cell function experiments showed that compared with the Control group, the proliferation ability of cells in the LPS group increased, senescence and apoptosis decreased. In the IL-4 group, cell proliferation was decreased, senescence and apoptosis were increased. After butyrate was added, cell proliferation ability of the LPS+butyrate group and the IL-4+butyrate group was decreased, and senescence and apoptosis were increased (Figures 5A–D). We then examined the expression of apoptosis, senescence signaling pathways related proteins and inflammatory cytokines (Bax, Bcl-2, p21, p16, IL-6, IL-12, TNF-α, and IL-10). As shown in Figure 5E, compared with the Control group, the expression of Bax, p21, and p16 in the LPS group were decreased, and the expression of Bcl-2, IL-6, IL-12, and TNF-α were increased. The expression of Bax, p21, and p16 increased in the IL-4 group, while the expression of Bcl-2, IL-6, IL-12, and TNF-α decreased. After butyrate was added, the expression of Bax, p21, and p16 increased in the LPS+butyrate group and the IL-4+butyrate group, while the expression of Bcl-2, IL-6, IL-12, and TNF-α decreased. These results indicated that butyrate inhibited rat myocardial fibroblasts activity.

### Butyric Acid Ameliorated Symptoms of MF in Rats

**Figure 6:**
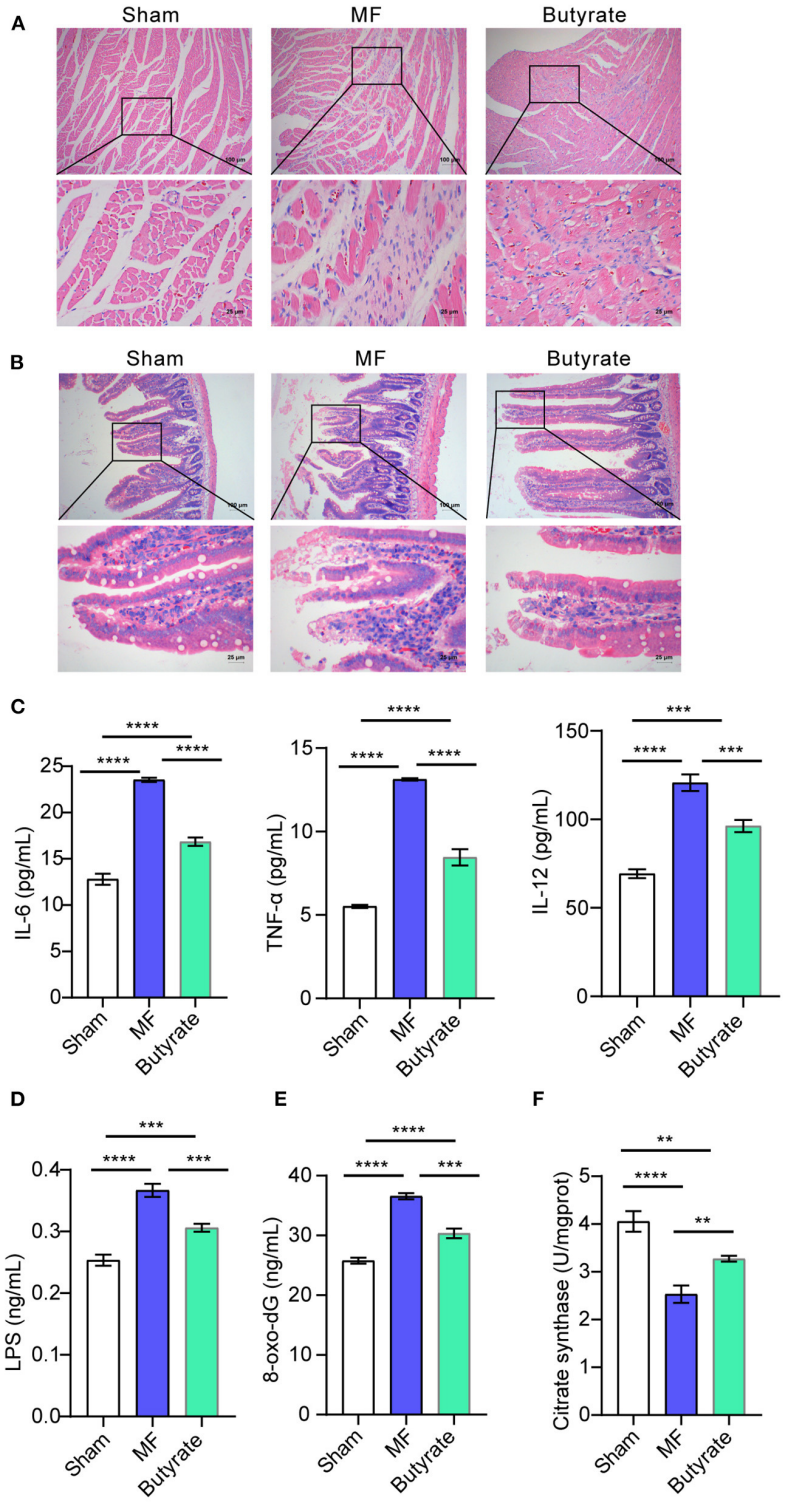
Butyric acid ameliorated symptoms of MF in rats. **(A)** HE staining was applied to stain the heart tissues. **(B)**. HE staining was performed to stain the gut tissues. **(C)** The levels of IL-6, TNF-α, and IL-12 in plasma were determined by ELISA. **(D–F)**. ELISA was used to detect the levels of LPS, 8-oxo-dG and CS. **P < 0.01, ***P < 0.001, ****P < 0.0001.

Finally, we studied the effect of butyric acid on MF in rats *in vivo*. HE staining of the heart tissue showed that the Sham group had tight tissue morphology, neatly arranged muscle cells, and relatively full nuclei. In the MF group, muscle cells were atrophy, and had more inflammatory cells, obvious inflammation, disordered tissue arrangement, and fibrosis symptoms. However, after butyric acid was used, MF symptoms were alleviated somewhat (Figure 6A). HE staining of the gut showed that the intestinal villi in the sham group were arranged neatly, while the inflammatory cells in the lamina propria of the MF group increased, the villi were arranged more disorderly, and the superficial mucosa was eroded. After the use of butyric acid, the inflammatory infiltration was reduced and the villi were aligned (Figure 6B). In addition, compared with the Sham group, the levels of pro-inflammatory factors (IL-6, IL-12, and TNF-α) in the MF group were increased. However, after using butyric acid, the levels of pro-inflammatory factors (IL-6, IL-12, and TNF-α) were decreased (Figure 6C). Consistent with this, compared with the Sham group, LPS, 8-oxo-dG and CS levels in the MF group were increased, while LPS, 8-oxo-dG and CS levels were decreased after butyric acid was used (Figures 6D–F). These results suggested that butyric acid could protect mitochondria and improve the symptoms of MF in rats.

## Discussion

MF is an inevitable pathological process in the end stage of many cardiovascular diseases, often leading to severe cardiac dysfunction and even death (24). Treatments to alleviate MF have yet to be developed to effectively protect the heart. Therefore, in this study, we constructed a rat model of MF and fed rats with butyric acid. 16S rRNA sequencing was used to analyze the gut microbiota characteristics of the Sham group and MF group. In addition, influences of butyrate on M1/M2 polarization of rat macrophages, mitochondrial function and rat myocardial fibroblasts were discussed *in vitro* experiments. Our results suggested that butyric acid ameliorated MF by regulating M1/M2 polarization of macrophages and promoting recovery of mitochondrial function. At present, there is no reported study on the mechanism of butyric acid regulating the mitochondrial function of macrophage polarization in the MF, which is also the innovation of this study.

In recent years, more and more evidences show that gut microbiota and its metabolites play a key role in the occurrence and development of cardiovascular diseases such as atherosclerosis, hypertension, heart failure, atrial fibrillation, and MF. Trillions of bacteria reside in the gastrointestinal tract, metabolizing nutrients into SCFA, etc. (25). *Desulfovibrionaceae* and *Helicobacteraceae* have been reported in the heart-related diseases. A previous study showed that combined consumption of beef-based cooked mince and sucrose could stimulate oxidative stress, heart hypertrophy and colonic outgrowth of *Desulfovibrionaceae* (26). In addition, filled with multi-walled carbon nanotubes in the trachea, a standard fine particle, could alter gut microbiota, increase the abundance of *Helicobacteraceae*, and exacerbate doxorubicin-induced cardiotoxicity in mice (27). Our study is consistent with the previous results that the abundance of *Desulfovibrionaceae* and *Helicobacteraceae* in the MF group was increased compared with the Sham group. Butyric acid, a SCFA produced daily by gut microbiota, has been shown to benefit cardiovascular disease models. It has been reported that gut microbiota affected the acetylation level and tissue repair process after myocardial infarction by affecting the production of butyric acid (28). Previous studies have shown that butyrate intervention decreased potentially pathogenic species, including *Desulfovibrionaceae*, which was a potential group of endotoxin producers (29, 30). Jena PK et al. reported that probiotics VSL#3 reconstructed the gut microbiota by reducing *Bacteroidaceae, Porphyromonadaceae*, and *Helicobacteraceae* as well as increasing *Lachnospiraceae*. Further, VSL#3 enriched the abundance of *Ruminococcus* and *Faecalibacterium*, which generate butyrate, at the genus level (31). Through Pearson correlation coefficient analysis, we found that butyric acid was significantly negatively correlated with *Helicobacteraceae* and *Desulfovibrionaceae*. Therefore, we were interested in butyrate changes. We found that the content of butyric acid in MF group was decreased compared with the Sham group. Therefore, we speculate that butyric acid supplementation might improve MF.

After tissue injury, monocytes and macrophages that reside in tissues undergo significant phenotypic and functional changes, which are key regulatory factors of tissue repair, regeneration and fibrosis (32). In altered myocardial microenvironment, cardiac macrophages exhibit different phenotypes (M1 or M2) and functions (pro-inflammatory or anti-inflammatory) and subsequently exacerbate or resolve inflammation in the infarcted heart. The regulation of macrophage polarization is related to the quality of myocardial infarction and the result of cardiac healing (33). Macrophage polarization determines the transition from inflammatory stage to inflammatory subside stage after myocardial infarction (34). Promoting anti-inflammatory M2-like polarization of macrophages after MI could improve cardiac injury after myocardial infarction (35, 36). In this study, LPS and IL-4 were used to stimulate rat macrophage RMa-bm, respectively, and butyrate was added. We found that in LPS (M1) and IL-4 (M2) polarization, butyrate inhibited M1 and promoted M2 polarization. Mitochondria are involved in many cellular processes and their main role is to produce cellular energy. They constantly undergo fission and fusion, and these canceling processes are in strict equilibrium (37). The heart is a highly oxidized tissue, so mitochondria play an important role in maintaining optimal cardiac function. Both human and experimental animal failing hearts have abnormal mitochondrial structure and function, which are manifested as hyperplasia, smaller organelles, reduced ATP synthesis rate and increased ROS formation, etc. (38). It has been reported that mitochondrial fission played a key role in the progression of obesity-induced MF (39). Studies have shown that the novel butyric acid derivative phenylalanine butyamide could protect the cardiotoxicity of doxorubicin by reducing oxidative stress and improving mitochondrial function (40). Our results showed that butyrate may promote mitochondrial function recovery by regulating M1/M2 polarization of macrophages. The results of feeding butyric acid in MF rats also showed that butyric acid could protect mitochondria and improve the symptoms of MF rats.

The myocardial microenvironment includes cardiomyocytes, fibroblasts, and macrophages, which regulate remodeling after myocardial infarction. Myocardial fibroblasts are involved in many aspects of cardiac function and pathophysiology. Typical roles of macrophages in mediating fibrotic responses after heart attack include ECM transformation and activation of myocardial fibroblasts to initiate collagen deposition (41). Studies have shown that BIO regulated molecular crosstalk between myocardial fibroblasts and differentiated macrophages, inducing polarization to an anti-inflammatory M2 phenotype (42). When diastolic dysfunction occurs, cardiac macrophages produce IL-10, which activates fibroblasts and stimulates collagen deposition, leading to impaired myocardial relaxation and increased myocardial stiffness (43). Jung et al. (44) reported that IL-10 treatment increased fibroblast activation (proliferation, migration, and collagen production) *in vivo*, and this effect was directly or indirectly affected by M2 polarization of macrophages. Nudelman V et al., reported that the anticancer prodrug AN-7 released the histone deacetylase inhibitor butyric acid and caused histone hyperacetylation upon metabolic hydrolysis. However, AN-7 could reduce hypoxia-induced mitochondrial damage and cell death of cardiomyocytes, reduce hydrogen peroxide damage of H9c2 cells, and increase cell damage and death of hypoxic myocardial fibroblasts (45). These results suggested that butyric acid may increase cell damage and death of hypoxic cardiac fibroblasts. Our research was consistent with that. Results of cell co-culture showed that after butyrate was added, cell proliferation ability was decreased, and senescence and apoptosis were increased, which indicated that butyrate inhibited the activity of rat myocardial fibroblasts. However, our study did not examine changes in gut microbiota after butyrate treatment, which is our limitation. Due to time and financial constraints, we cannot solve this problem very well. In the future, we will perform 16S rRNA sequencing on the Sham, MF and Butyrate groups to examine changes in gut microbiota.

## Conclusion

In this study, we first confirmed that the content of butyric acid decreased in the MF group. In addition, the mechanism involved in butyric acid was preliminarily explored. We also found that butyrate regulated the mitochondrial function of macrophage polarization in the MF by *in vivo* and *in vitro* experiments. Our study provides an important clue for further understanding the mechanism of the occurrence and development of MF, and provides a reference and basis for the clinical treatment of MF.

## Data Availability Statement

The datasets presented in this study can be found below: https://www.ncbi.nlm.nih.gov/bioproject/PRJNA811334.

## Ethics Statement

The animal study was reviewed and approved by the IRB of Third Xiangya Hospital, Central South University (2018-S259).

## Author Contributions

XL wrote the paper. XL, RL, NY, XZ, and JL performed the experiments and analyzed the data. WJ conceived and designed the experiments. All authors agree to be accountable for the content of the work.

## Funding

The study was supported by the National Natural Science Foundation of China Projects (NO. 81800271), the Natural Science Foundation of Hunan Province (NO. 2019JJ50920), and Key Research and Development program of Hunan Province (NO. 2022SK2029).

## Conflict of Interest

The authors declare that the research was conducted in the absence of any commercial or financial relationships that could be construed as a potential conflict of interest.

## Publisher's Note

All claims expressed in this article are solely those of the authors and do not necessarily represent those of their affiliated organizations, or those of the publisher, the editors and the reviewers. Any product that may be evaluated in this article, or claim that may be made by its manufacturer, is not guaranteed or endorsed by the publisher.

## Supplementary Material

Supplementary Figure 1 Flow cytometry results about influences of butyrate on M1/M2 polarization of rat macrophages RMa-bm. **(A)** Flow cytometry results about CD86 (+), CD80 (+), CD64 (+), and CD16/32 (+) positive cell rates in the Control, LPS, and LPS+butyrate groups, respectively. **(B)** Flow cytometry results about CD163 (+) and CD206 (+) positive cell rates in the Control, IL-4, and IL-4+butyrate groups.
