# Supplementary figures and images for: Butyric Acid Ameliorates Myocardial Fibrosis by Regulating M1/M2 Polarization of Macrophages and Promoting Recovery of Mitochondrial Function
Source: Front Nutr. 2022 May 18;9:875473. doi: 10.3389/fnut.2022.875473 (PMC9159497; doi:10.3389/fnut.2022.875473)

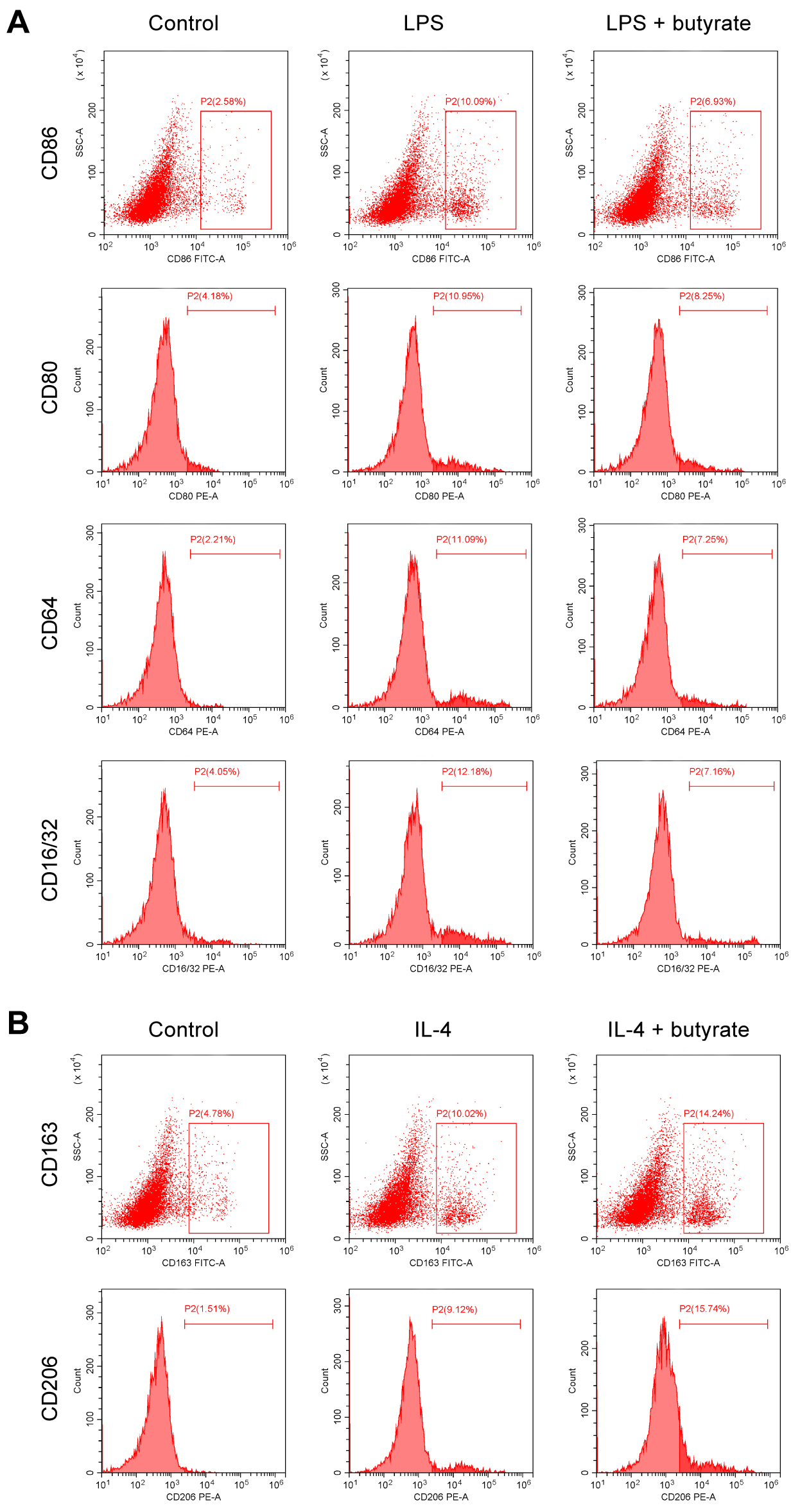

Supplement: Supplementary Figure 1 — Flow cytometry results about influences of butyrate on M1/M2 polarization of rat macrophages RMa-bm. (A) Flow cytometry results about CD86 (+), CD80 (+), CD64 (+), and CD16/32 (+) positive cell rates in the Control, LPS, and LPS+butyrate groups, respectively. (B) Flow cytometry results about CD163 (+) and CD206 (+) positive cell rates in the Control, IL-4, and IL-4+butyrate groups. [file Image_1.JPEG]
